# 839. Artificial Intelligence-enhanced Multispectral Imaging for Burn Wound Assessment: A Multi-centre UK Evaluation Update

**DOI:** 10.1093/jbcr/irag033.278

**Published:** 2026-04-06

**Authors:** Poh (Leslie) Tan, Shreyas V Supparamaniam, Zeeshan Sheikh, Christopher J Lewis

**Affiliations:** Royal Victoria Infirmary, Newcastle, Newcastle upon Tyne, England; Royal Victoria Infirmary, Newcastle, Newcastle upon Tyne, England; Manchester NHS Foundation Trust, Manchester, England; Northern Regional Burn Centre, Newcastle Upon Tyne, England

## Abstract

**Introduction:**

Real-world validation in large patient cohorts is essential to establish the effectiveness of emerging diagnostic technologies. In our earlier evaluation of 34 patients and 73 burn images, AI-enhanced multispectral imaging (MSI) achieved 95.3% accuracy and was used across multiple clinical environments, demonstrating potential to improve diagnostic accuracy and reproducibility. We now present updated findings from an expanded multicentre UK product evaluation, utilising a larger dataset and refined analytical methods.

**Methods:**

We continued our investigation with a multicentre, prospective product evaluation in two tertiary-level burn centres in the UK, assessing adults (≥18 years) with superficial to full-thickness burns who were not requiring or declining surgery. Burns involving the face, neck, hands, or feet were excluded, as these are non-validated anatomical sites. AI-generated predictions were compared with the 21-day clinical healing assessment, which served as the reference standard for evaluation. The primary outcome was the reliability and reproducibility of healing prediction, and the secondary outcome was the feasibility of the device. Burn images were analysed using ImageJ, and statistical analyses were performed in JASP.

**Results:**

Ninety-three patients and 132 burn images (24 million data points) were analysed. The mean age was 49 years, with an average total body surface area of 4.6%, and scalds being the most common mechanism (n = 47). The AI-MSI system achieved a sensitivity of 79.3% (95% CI 79.2–79.5%) and a specificity of 96.1% (95% CI 96.1–96.2%). Positive predictive value of 31.4% (95% CI 31.3–31.5%) and negative predictive value of 99.5% (95% CI 99.5–99.5%). The overall accuracy of the system was 95.7% (95% CI 95.7–95.8%). The device was portable and used in clinics, operating theatres, and emergency departments.

**Conclusions:**

This multicenter evaluation update demonstrates that AI-enhanced multispectral imaging can deliver consistent, high-accuracy burn depth assessment in various clinical settings. Beyond confirming diagnostic performance, the study highlights the potential of deploying the technology in real-world workflows, providing a foundation for its role in standardising burn assessment and supporting precision-guided treatment strategies.

**Applicability of Research to Practice:**

Translating these findings this longer-term analysis shows the AI-enhanced MSI can function as a practical adjunct to clinical assessment, offering reproducible and accurate depth evaluation across varied care settings. Wider clinical adoption will depend on extending validation to complex anatomical regions, paediatric cohorts, and outcome-linked studies, but this evaluation provides an important step toward standardising burn depth assessment through AI-enabled imaging.

**Funding for the study:**

N/A.

